# Limited Mechanistic Link Between the Monod Equation and Methanogen Growth: a Perspective from Metabolic Modeling

**DOI:** 10.1128/spectrum.02259-21

**Published:** 2022-03-03

**Authors:** Qusheng Jin, Qiong Wu, Benjamin M. Shapiro, Shannon E. McKernan

**Affiliations:** a Geobiology Group, University of Oregon, Eugene, Oregon, USA; University of Minnesota

**Keywords:** Monod equation, half-saturation constant, maximum growth rate, metabolic modeling, methanogenesis, microbial kinetics, specific affinity

## Abstract

The Monod equation has been widely applied as the general rate law of microbial growth, but its applications are not always successful. By drawing on the frameworks of kinetic and stoichiometric metabolic models and metabolic control analysis, the modeling reported here simulated the growth kinetics of a methanogenic microorganism and illustrated that different enzymes and metabolites control growth rate to various extents and that their controls peak at either very low, intermediate, or very high substrate concentrations. In comparison, with a single term and two parameters, the Monod equation only approximately accounts for the controls of rate-determining enzymes and metabolites at very high and very low substrate concentrations, but neglects the enzymes and metabolites whose controls are most notable at intermediate concentrations. These findings support a limited link between the Monod equation and methanogen growth, and unify the competing views regarding enzyme roles in shaping growth kinetics. The results also preclude a mechanistic derivation of the Monod equation from methanogen metabolic networks and highlight a fundamental challenge in microbiology: single-term expressions may not be sufficient for accurate prediction of microbial growth.

**IMPORTANCE** The Monod equation has been widely applied to predict the rate of microbial growth, but its application is not always successful. Using a novel metabolic modeling approach, we simulated the growth of a methanogen and uncovered a limited mechanistic link between the Monod equation and the methanogen’s metabolic network. Specifically, the equation provides an approximation to the controls by rate-determining metabolites and enzymes at very low and very high substrate concentrations, but it is missing the remaining enzymes and metabolites whose controls are most notable at intermediate concentrations. These results support the Monod equation as a useful approximation of growth rates and highlight a fundamental challenge in microbial kinetics: single-term rate expressions may not be sufficient for accurate prediction of microbial growth.

## INTRODUCTION

In 1942, the French scientist Jacques Monod introduced a single mathematical expression,

(1) $$\mu =\mu_{\max}\frac{C}{C\;+\;K_{\text{M}}}$$

and two parameters, maximum growth rate (*μ*_max_) and half-saturation constant (*K*_M_), to describe his discovery that specific rate (*μ*) of microbial growth, the relative rate of biomass increase (s^−1^), responds hyperbolically to the concentration (*C*) of limiting substrate (1, 2). This equation, named after him, is analogous to the Michaelis-Menten equation for enzymes, but it describes growth kinetics emerging from hundreds to thousands of enzymes. It enables the quantitative prediction of microbial population dynamics and has become a standard rate law for the analysis and modeling of microbial processes across disciplines, and a vital tool for addressing current environmental challenges, from biofuel production to contaminant remediation and global carbon cycling (3–5).

In response to the fundamental importance and wide application of the Monod equation, considerable attention has been directed towards deriving the rate law (6, 7). Some derivations have followed a reductionist approach, assuming a single rate-determining or rate-limiting enzyme (8, 9), focusing on specific cellular process such as substrate uptake and protein synthesis (7, 10), or reducing entire metabolism to a two-step or multistep linear process (11, 12). Others have built on analogs from familiar physics, including resistors in series (13), transition state theory (14), statistical quantum mechanics (15), and thermodynamics (16). These efforts simplified microbial metabolism to different extents and, as a result, masked the mechanistic link between the Monod equation and the microbial metabolic network.

A related point of discussion is that the Monod equation may represent an oversimplification of microbial growth. As early as the 1950s, experimentalists have reported that the equation did not always accurately reproduce the hyperbolic growth of laboratory cultures (17–19). Likewise, application to natural environments resulted in predictions which deviated from field observations by orders of magnitude (20). These observations challenged the application of the rate law and triggered an extensive search for alternative rate expressions, a topic still receiving considerable attention today (21, 22).

The discrepancy between model and observations also gave rise to the contention that the Monod equation might not reflect the metabolic complexity associated with microbial growth (3, 21). Similar to rate laws for abiotic chemical reactions (23), the Monod equation and other commonly used microbial rate laws employ single mathematical expressions with constant parameters. In contrast, hundreds to thousands of enzymes from the pathways of energy conservation, amino acid synthesis, signal transduction, and others work in concert to establish a metabolic reaction network that reproduces biomass. However, many physical laws, such as Newton’s three laws of motion and the ideal gas law, appear relatively simple, and simplicity alone is not sufficient to rule out a mechanistic link between the Monod equation and the metabolic network of microbial growth.

**FIG 1:**
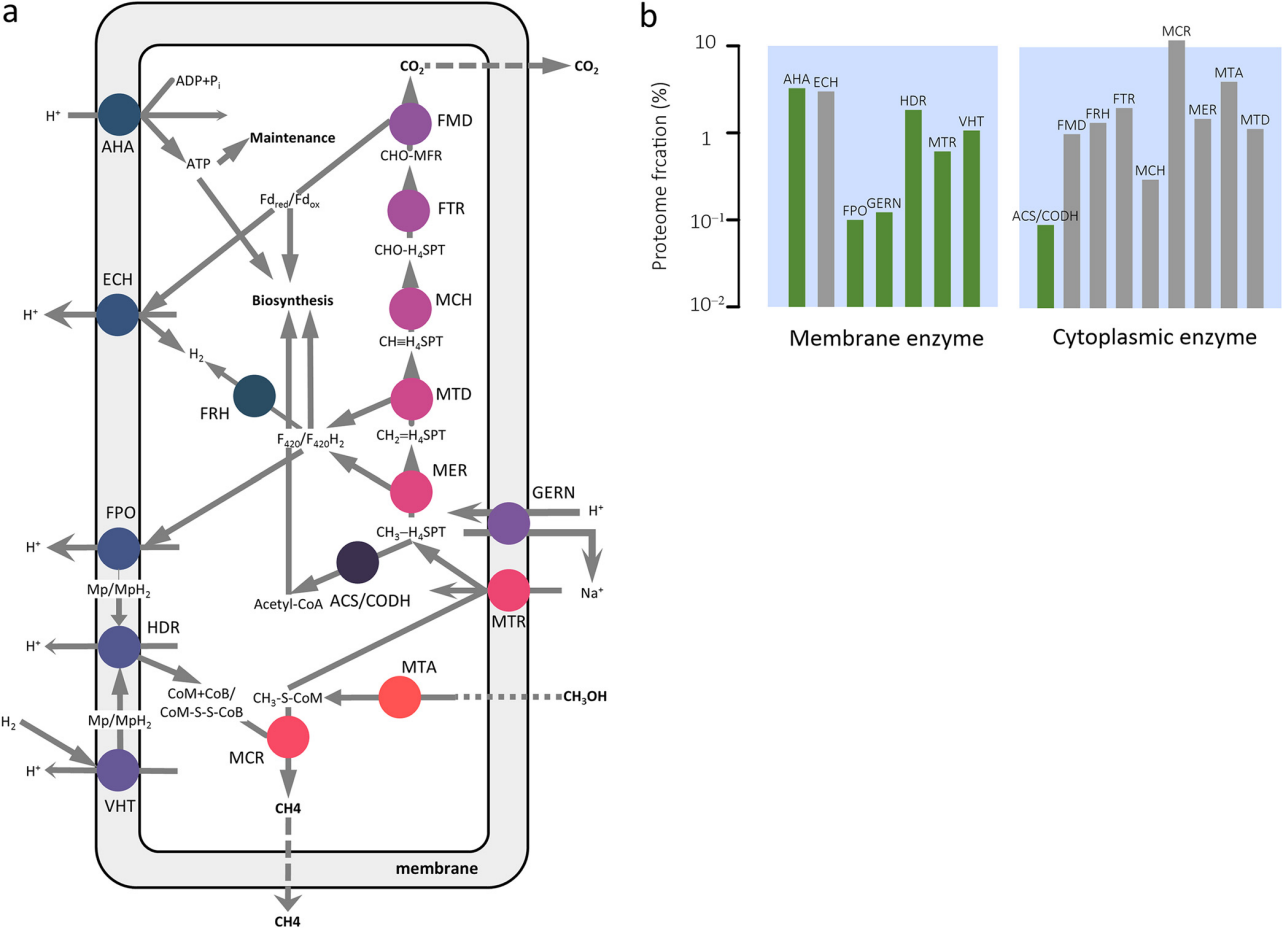
(a) A kinetic metabolic model of *M. barkeri* that focuses on the methanogenesis pathway. Methanol diffuses into the cytoplasm and is processed to synthesize ATPs, reduced cofactors, and acetyl-coenzyme A, which are then consumed by pseudo-reactions of maintenance and biomass synthesis. Dashed and solid arrows indicate diffusion and biochemical reactions, respectively; circles represent enzymes. (b) Proteome fractions of enzymes applied in simulating growth. Green indicates enzyme abundances estimated by optimization, and gray indicates those obtained from *in vitro* cell-free lysates (see Supplementary Dataset S1). ACS/CODH, acetyl-CoA synthase/carbon monoxide dehydrogenase; AHA, ATP synthase; ECH, energy-converting ferredoxin-dependent hydrogenase; FMD, formylmethanofuran dehydrogenase; FPO, F_420_ dehydrogenase; FRH, F420-reducing hydrogenase; FTR, formylmethanofuran-tetrahydromethanopterin N-formyltransferase; GERN, sodium/proton antiporter; HDR, heterodisulfide reductase; MCH, methenyltetrahydromethanopterin cyclohydrolase; MCR, methyl-coenzyme M reductase; MER, 5,10-methylenetetrahydromethanopterin reductase; MTA, methanol:coenzyme M methyltransferase; MTD, methylenetetrahydromethanopterin dehydrogenase; MTR, methyl-H_4_SPT:coenzyme M methyltransferase; VHT, methanophenazine-dependent hydrogenase; CoA, coenzyme A; CH_3_CO-CoA, acetyl-coenzyme A; CoB, coenzyme B; CoM, coenzyme M; CoB-S-S-CoM or hsfd, mixed disulfide of CoB and CoM; F_420_/F_420_H_2_, oxidized and reduced cofactor F_420_, respectively; Fdox/Fdred, oxidized and reduced ferredoxin, respectively; Mp/MpH_2_, oxidized and reduced methanophenazine; CHO-MF, formyl-methanofuran; H_4_SPT, tetrahydrosarcinapterin; CHO-H_4_SPT, formyl-H_4_SPT; CH≡H_4_SPT, methenyl-H_4_SPT; CH_2_=H_4_SPT, methylene-H_4_SPT; CH_3_-H_4_SPT, methyl-H_4_SPT; CH_3_-CoM, methyl-coenzyme M.

Here, we seek to explore the mechanistic link between the Monod equation and microbial growth by using metabolic modeling. For this purpose, we assemble a kinetic metabolic model that features catabolic pathways, including substrate uptake and the production of ATPs, reducing equivalents, and carbon precursors, and couples production fluxes to biomass synthesis according to the stoichiometric model of genome-scale metabolic reactions (Fig. 1a). Ideally, we should simulate growth from enzyme kinetics, an approach that would require the kinetic information of every enzyme involved in cell reproduction. Our kinetic/stoichiometric-hybrid approach represents a compromise between the paucity of kinetic data for biosynthesis enzymes and the desire to simulate essential metabolic features that shape growth phenotypes. We analyze the model using metabolic control analysis (MCA), a sensitivity analysis framework for evaluating the extent to which emergent properties of a metabolic network as a whole are affected by small changes in the properties of its components (24, 25). This approach allows us to untangle the complexity of the control of growth rate by enzymes and metabolites, and to uncover emergent network properties that bear out the hyperbolic growth phenotype and the physical meanings of microbial kinetic parameters.

We simulate microbial growth using *M. barkeri* as a model system. Our preference for *M. barkeri* stems from extensive laboratory studies of its biochemistry, genetics, metabolism, and physiology (26, 27), and their results serve as baselines for constructing and validating our modeling approach. Moreover, methanogenic growth produces methane, a potent greenhouse gas, and hence is of environmental significance. We focus on methylotrophic methanogenesis,

(2) $$\frac{4}{3}\text{methanol}=\frac{2}{3}{\text{H}}_{2}\text{O}\;+\;\frac{1}{3}\;{\text{CO}}_{\text{2}}\;+\;{\text{CH}}_{\text{4}}$$

a process that has recently been recognized as one of the dominant methanogenesis pathways in marine sediments (28, 29). Our key results include (i) a hybrid computational framework that predicts methanogen growth rate on the basis of the stoichiometry of genome-scale metabolic reactions and the kinetics of methanogenesis enzymes, (ii) a new interpretation of the hyperbolic pattern of microbial growth, (iii) the limited link between the Monod equation and methanogen metabolic network via rate-determining enzymes and metabolites, and (iv) the amendment of the Monod equation with a Gaussian error function that improves the prediction of growth rates.

## RESULTS

### Model construction.

We built a kinetic metabolic model of *M. barkeri* growing on methanol and calibrated the model to represent the metabolic state of the organism acclimating to typical laboratory conditions (37 °C, pH 7, and anoxic mineral medium with 100 mM methanol as the sole carbon source) (26). This model treats *M. barkeri* as a spherical cell of two compartments, the cytoplasm covered by the membrane, and allows methanol, dissolved CO_2_, and CH_4_ to diffuse freely between the extracellular environment and the cell (Fig. 1). The model uses 9 enzymes in the cytoplasm and 7 enzymes associated with the membrane to represent how the methanogenesis pathway processes methanol to CO_2_ and CH_4_ and, at the same time, produces ATPs, carbon precursors, and reduced redox cofactors. To relate pathway fluxes to growth rate according to the Herbert-Pirt equation (30), the model also includes a hypothetical reaction of ATP hydrolysis to account for biomass maintenance, and a pseudo-reaction that produces biomass from ATPs, reduced cofactor F420 and ferredoxin, and acetyl coenzyme A (acetyl-CoA, CH_3_CO-CoA). Taken together, a total of 21 reactions consume and produce 35 metabolites.

Compared to the kinetic models developed for *M. acetivorans* (31), Escherichia coli (32), and yeast glycolysis (33, 34), our *M. barkeri* model is unique in the following aspects:

First, our model explicitly tracks energy fluxes through the metabolic network. To this end, the model simulates the buildup and consumption of membrane electrochemical potential. It also computes reaction velocity by using the generalized reversible Michaelis-Menten equation to account for reaction thermodynamics (35). These treatments are necessary, considering that metabolic reactions may proceed close to thermodynamic equilibrium and hence their rates may be limited by thermodynamics (36, 37).

Second, we derived the stoichiometry of the pseudo-biomass reaction from the *M. barkeri* genome-scale metabolic model by performing flux balance analysis (FBA) (38, 39). The FBA results show that synthesizing 1 g of biomass consumes 0.14 mol ATP, 1.1 × 10^−2^ mol reduced ferredoxin, 1.1 × 10^−2^ mol reduced F420, and 1.4 × 10^−2^ mol acetyl coenzyme A. By including the pseudo-biomass reaction, we constrained the kinetic model with the principle of mass balance at the genome-scale.

Last, we estimated the concentrations of membrane enzymes by using optimization. Enzyme concentrations are required to compute the velocities of enzyme reactions, but the abundances of most of the membrane enzymes have yet to be determined experimentally. As an alternative, we estimated their concentrations by maximizing the growth rate of *M. barkeri* under typical laboratory conditions. The results are shown in Fig. 1b, where the membrane enzymes have mass fractions of the proteome ranging from 0.1% to 3.3%, with a median value of 1.1%. These features enable us to compute growth rates from the properties and interactions of metabolic reactions without imposing *ad hoc* constraints on methane production or methanogen growth, and allow us to simulate methanogen metabolism limited by energy sources, a condition prevailing in both bioreactors and natural environments.

### Model validation.

**FIG 2:**
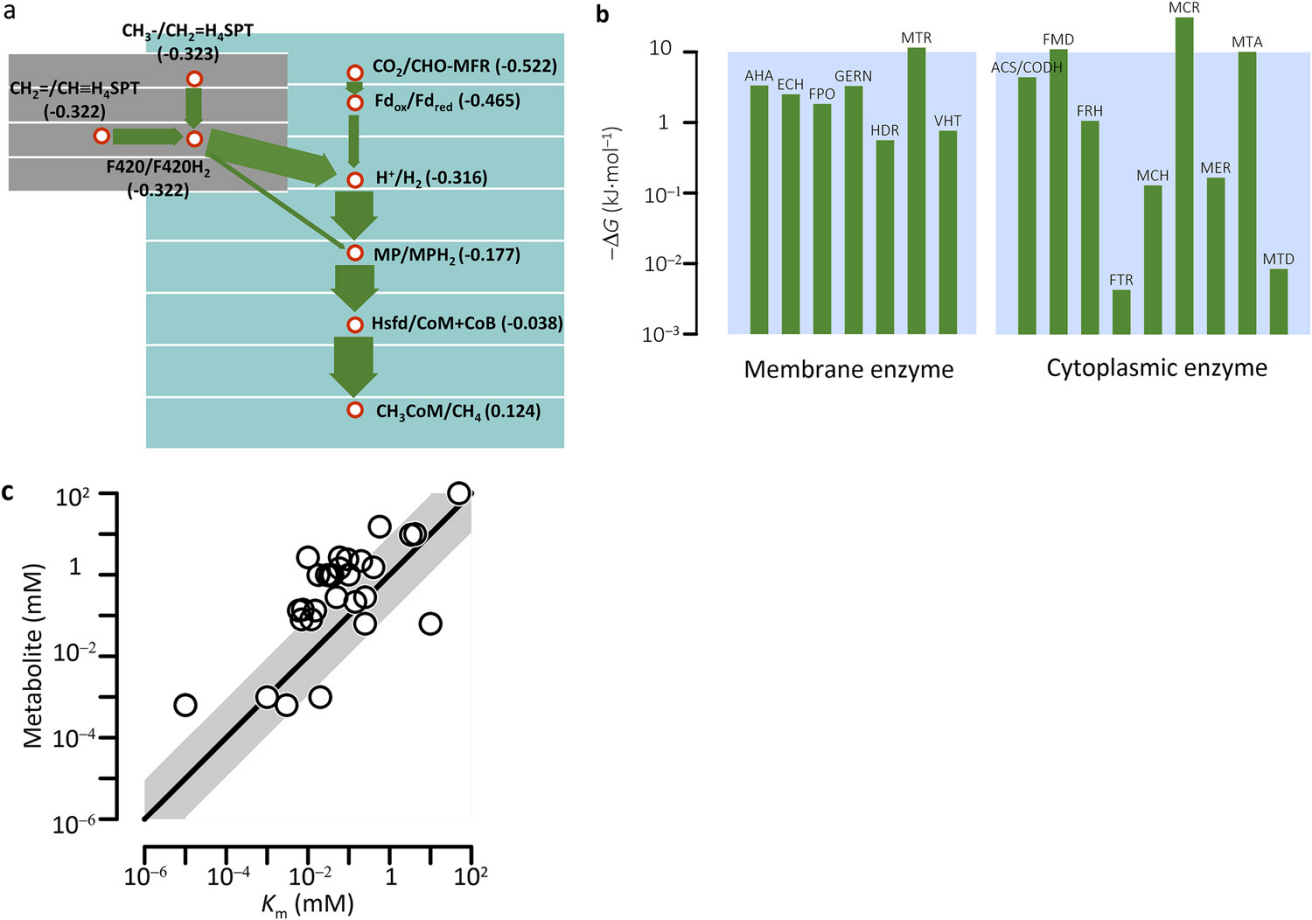
Kinetic metabolic model reproduces independent experimental observations. (a) Electron fluxes from the oxidation to the reduction of the methyl-group in methanol. Values in parentheses show reduction potentials (V); arrow widths indicate electron fluxes relative to the flux of the reduction of methyl-coenzyme M to methane (i.e., 3.6 × 10^−18 ^mol · s^−1^). (b) Gibbs free energy (Δ*G*) is unevenly distributed among enzyme reactions. (c) 81% of metabolites have concentrations greater than the respective Michaelis constants (*K*_m_). Solid line shows the 1:1 ratio; shaded area covers up to 10-fold deviations from the 1:1 ratio. See the Fig. 1 legend for definitions of abbreviations.

To validate the metabolic model, we simulated the metabolism of *M. barkeri* growing under typical laboratory conditions and compared the simulation results at steady-state to the independent experimental observations that had been excluded from the model construction. For example, the simulated membrane potential is 135 mV, close to the experimentally determined value of 130 mV (40). The simulated H_2_ concentration is 0.7 μmolal, also close to the laboratory observation (i.e., 0.2 μmolal) (41). In addition, H_2_ and cofactor F420 share similar reduction potentials (Fig. 2a), which has been observed in laboratory experiments (42). Of the electron fluxes from the oxidation to the reduction of the methyl-group in methanol, 98% are carried by the production and consumption of H_2_, and cofactor F420 oxidation accounts for the remaining 2% (Fig. 2a), consistent with the dominant role of hydrogen cycling detected by laboratory experiments (43, 44).

The model reproduces two patterns documented by metabolomic studies of E. coli (36, 45). First, free energy is unevenly distributed among metabolic reactions, ranging from −31 kJ · mol^−1^ to <−0.01 kJ · mol^−1^ (Fig. 2b). Second, the majority (i.e., 84%) of metabolites have concentrations greater than their respective Michaelis constants (Fig. 2c).

The model predicts that at 100 mM methanol, *M. barkeri* laboratory cultures produce 0.21 CO_2_ and 0.67 CH_4_ molecules by consuming one molecule of methanol. These values are smaller than the theoretical stoichiometric coefficients of the methanogenesis reaction (0.25 CO_2_ and 0.75 CH_4_, respectively, see equation 2), but close to those determined by the radioactive tracer technique (e.g., 0.21 CO_2_ and 0.64 CH_4_) (46, 47). This can be accounted for by the methanol consumption in the production of reduced cofactors and acetyl coenzyme A, the metabolites required by biosynthesis (48).

**FIG 3:**
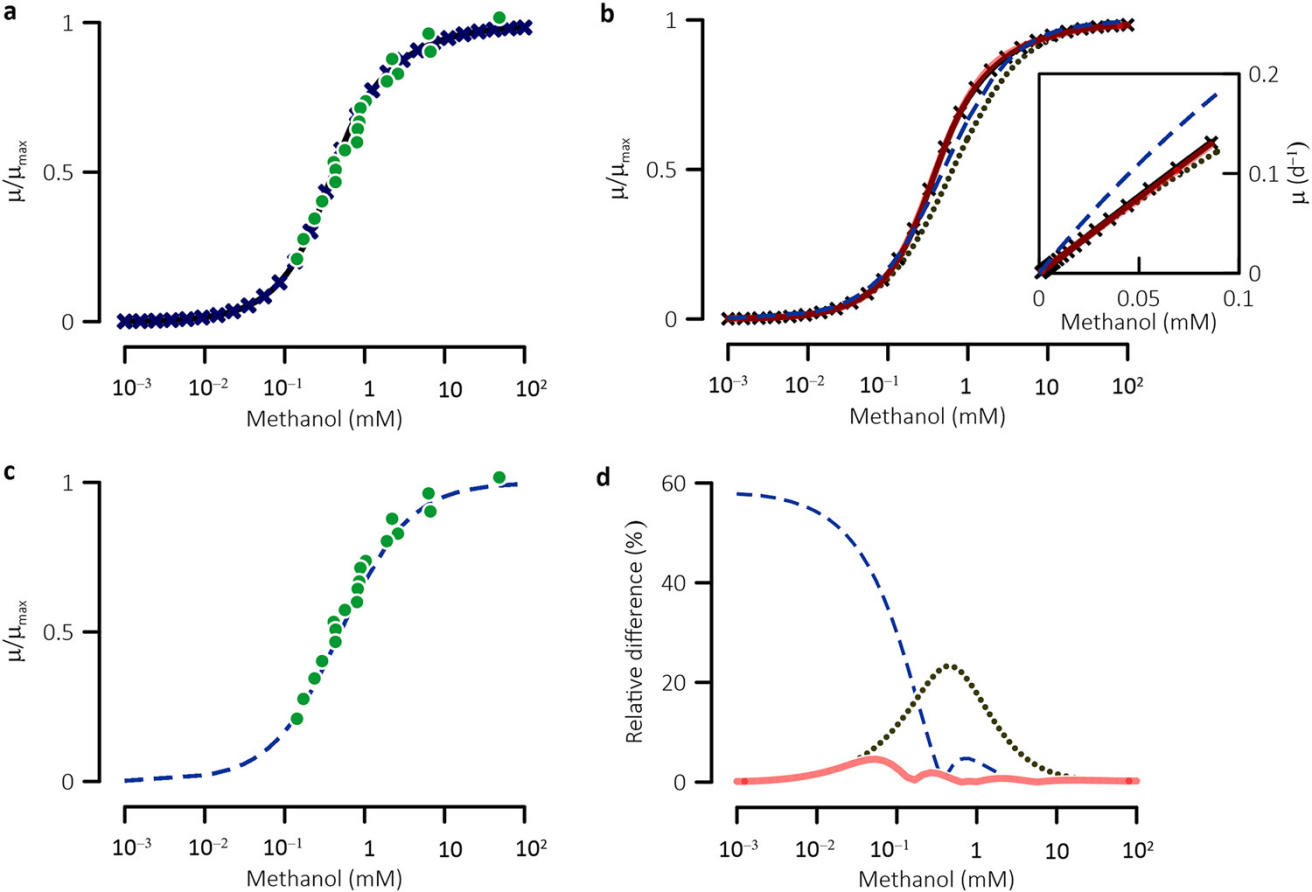
(a, b, and c) Specific growth rate (*μ*) varies hyperbolically with external methanol concentration. (d) Variations with methanol concentration in relative difference between the Monod equation and the simulation results. Insert in panel b shows rates at relatively low methanol concentrations; data points in green represent experimental observations of Daußmann et al. (49); lines with “X” data markers are specific growth rates, sums of net growth rates, and the maintenance rate, obtained from metabolic simulation; blue dash lines represent the results of the Monod equation (equation 1), evaluated using the maximum growth rate and half-saturation constants determined phenomenologically from the simulation results; dark dotted lines are calculated using equation 8 and the simulation-derived maximum growth rate and specific affinity; red solid lines represent the Monod equation amended with a Gaussian function (equation 11).

The model also predicts that the growth rate of *M. barkeri* laboratory cultures varies hyperbolically with the external methanol concentration. This prediction was obtained by simulating the growth of the laboratory cultures at external methanol concentrations ranging from 1 μM to 100 mM. The results fit to the laboratory observations of Daußmann et al. (49), with an *R*^2^ of 0.96 (Fig. 3a). Three parameters have been applied to characterize hyperbolic growth relationships: maximum growth rate (*μ*_max_), half-saturation constant (*K*_M_), and specific affinity (α). Following the phenomenological interpretation of these parameters, we approximated *μ*_max_ with the growth rate at 1 M methanol, determined *K*_M_ as the methanol concentration that drives growth at half of *μ*_max_, and estimated *α* as the slope of the rate increase at methanol concentrations <10 μM. The results are 1.0 d^−1^ for *μ*_max_, 0.4 mM for *K*_M_, and 1.6 ± 0.0 × 10^3^ M^−1^ · d^−1^ for *α*. The values of *μ*_max_ and *K*_M_ are close to those obtained from the laboratory, determined to be 1.0 ± 0.5 d^−1^ and 0.4 ± 0.2 mM, respectively (see Text S1 in the supplementary material), but the specific affinity has yet to be analyzed experimentally. Combining these tests, we concluded that our model reproduces previous laboratory observations across different scales and can be applied to investigate the kinetics of methanogen growth.

### Sensitivity analysis.

To uncover the mechanistic link between the Monod equation and the metabolic network of *M. barkeri*, we performed sensitivity analysis on the metabolic model using MCA. We computed both the scaled flux control coefficients of enzymes and the scaled flux response coefficients of metabolites (50). These coefficients measure the fractional change in growth rate by a fractional change in the concentration of an enzyme or a metabolite. A coefficient near 0 occurs when a network component places little influence on growth rate, whereas a value near 1 indicates that a component is paramount in determining growth rate.

We first analyzed the control of growth rate by methanogenesis enzymes. The role of enzymes in shaping growth rates has been widely appreciated, but the mechanistic underpinning of growth-rate control remains controversial. In particular, traditional kinetic theories assume that growth rate is determined by a single ‘pacemaker’ or rate-determining enzyme (7, 9), while both metabolic control theory and metabolic engineering emphasize that chemical fluxes through a microbial metabolic network, and hence growth rate, are controlled by all network enzymes, and that the flux controls vary with environmental conditions (24, 25, 51).

**FIG 4:**
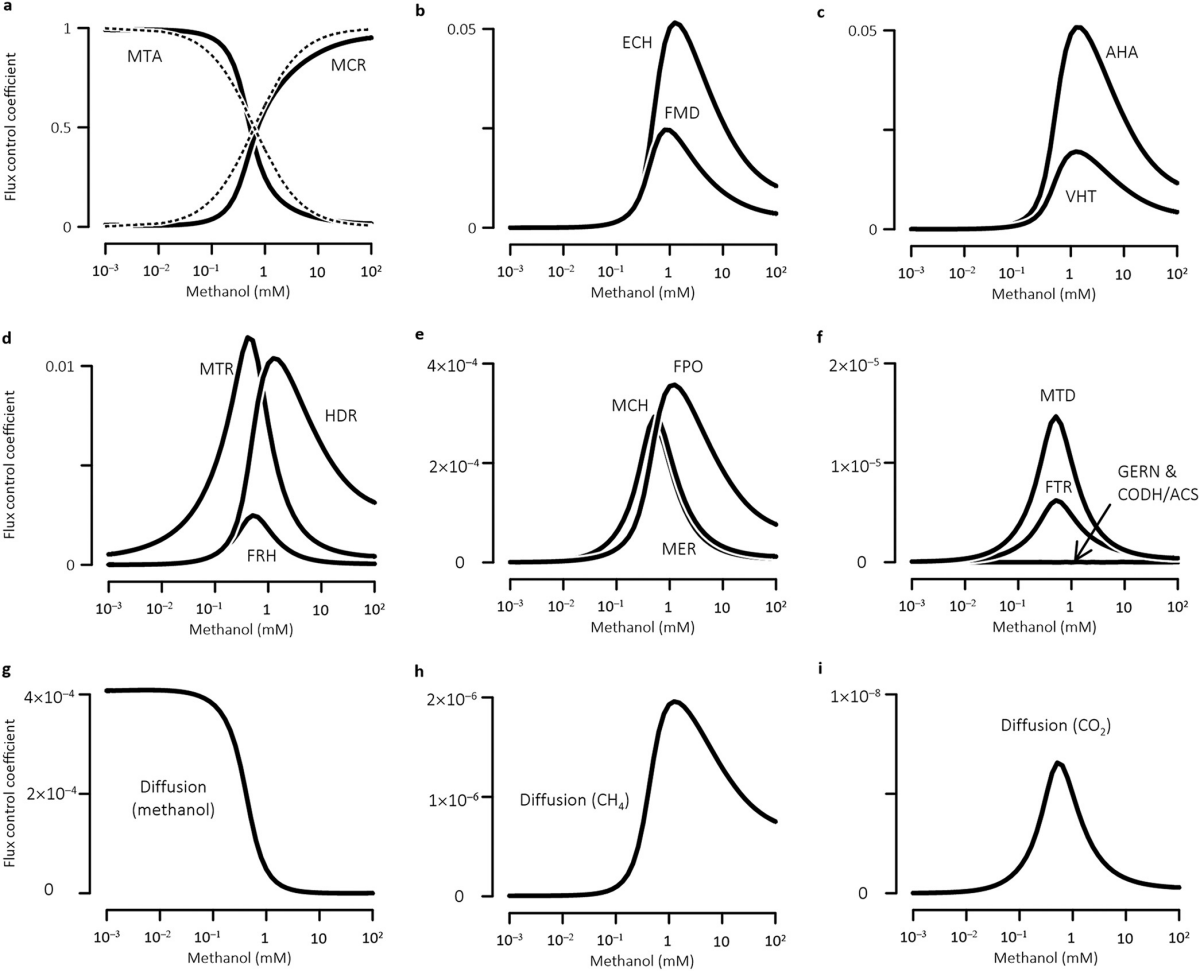
Scaled flux control coefficients of different enzymes (a to f) and diffusion processes (g to i) vary with ambient methanol concentrations to different extents. Solid lines are the results of MCA; dashed lines represent the conditions (equations 9 and 10) under which growth rate follows the Monod equation (equation 1). See the Fig. 1 legend for definitions of abbreviations.

The flux control coefficients obtained at various external methanol concentrations reveal that growth-rate controls by different enzymes shift with methanol concentrations to different extents (Fig. 4). Specifically, the scaled control coefficient of methanol:coenzyme M methyltransferase (MTA) stays close to 1 (i.e., >0.9) at very low methanol (<0.2 mM), while that of methyl-coenzyme M reductase (MCR) rises above 0.9 at >15 mM methanol. At intermediate concentrations (0.2 ∼ 15 mM), the coefficients of the two enzymes vary in opposite directions, crossing over at a methanol concentration of ∼0.6 mM. In contrast, the coefficients of the other enzymes remain <0.02 at very high and low methanol concentrations, and reach their maximum values (<0.06) between 0.6 and 1.2 mM methanol. In addition, diffusive methanol uptake and the release of CO_2_ and CH_4_ have control coefficients of <10^−3^.

From the flux control coefficients, we can approximate MCR and MTA as the rate-determining enzymes at >15 mM and <0.2 mM methanol, respectively. The dominant control by MCR is consistent with the long-standing hypothesis that MCR is a rate-controlling enzyme (52, 53), but this hypothesis may only be valid at very high methanol concentrations, such as those in laboratory bioreactors. The significant control of MTA at <0.2 mM methanol resonates with the observation that nutrient permeases and transporters dominate the control of metabolic fluxes at low substrate levels (54–56). Methanol diffuses freely through the membrane and does not require a permease. Also, diffusive methanol uptake does not significantly affect growth rate, which is in agreement with the previous assessment (3). Instead, methanol consumption by MTA in the cytosol determines the uptake flux of methanol, and hence the growth rate. Finally, these results are consistent with laboratory reports that microbial growth on other nutrients, such as glucose and acetate, is controlled by different enzymes at high and low substrate concentrations (54, 57).

We then analyzed control by metabolites. Metabolites regulate metabolic fluxes, and hence growth rate, on different levels, from specific allosteric regulation to global transcriptional and translational regulation (58, 59). Here, we focused on the control exerted by the reactants and products of metabolic reactions. In the methanogenesis pathway, most metabolites contain chemical moieties and, because of the conservation of chemical moieties, their concentrations are not independent (50). For example, over short time scales, the total concentrations of coenzyme M and methyl-coenzyme M do not change. Likewise, the total concentrations of reduced and oxidized ferredoxin remain constant. Therefore, we calculated the flux response coefficients of the total concentrations of chemical moieties.

**FIG 5:**
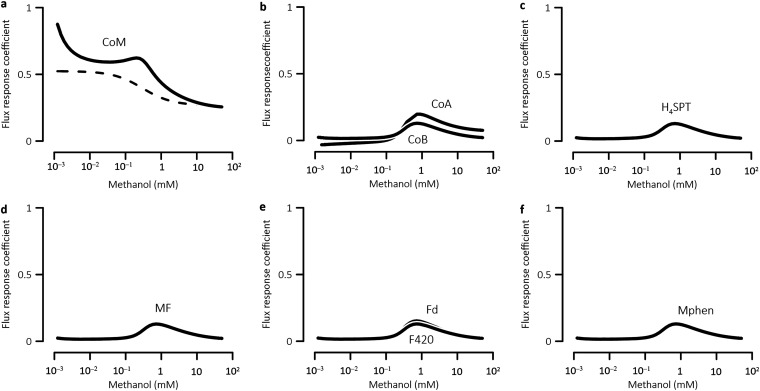
Scaled flux response coefficients of different chemical moieties vary with ambient methanol concentrations to different extents. Solid lines are the results of MCA; dashed line is the result obtained numerically from equations 3 to 6. See the Fig. 1 legend for definitions of abbreviations.

Fig. 5 shows how flux response coefficients vary with methanol concentrations. The coefficient of coenzyme M moiety is 0.9 at 1 μM methanol, and decreases to 0.3 at 100 mM methanol. In comparison, the remaining moieties have relatively low coefficients, i.e., <0.2. Their coefficients vary with methanol concentration according to bell-shaped curves, with values of <0.02 at <0.2 mM and >15 mM concentrations, and reach maximum values at intermediate concentrations. These results suggest that coenzyme M moiety dominates the control of growth rate across different methanol concentrations, while controls by the other moieties are most significant at intermediate concentrations. Moreover, around 1 μM, coenzyme M moiety can be approximated as a rate-determining moiety.

The remarkable control by coenzyme M moiety can be explained by its interactions with MCR and MTA, the two rate-determining enzymes. MCR and MTA consume methyl-coenzyme M and coenzyme, respectively, the two metabolites that contain coenzyme M moiety. Therefore, increases in moiety concentration speed up the reactions of MCR and MTA and hence, the growth rate. The association of rate-determining metabolites with rate-determining enzymes also appears in other metabolic processes. For example, while methionine is the rate-determining metabolite of ethylene production, methionine-consuming S-adenosylmethionine synthetase is the rate-determining enzyme of the process (60). As a second example, cysteine is the rate-limiting metabolite for glutathione biosynthesis, and the rate-determining enzyme is glutamate cysteine ligase (61). Combining the MCA results, we conclude that there are two rate-determining steps in the methanogenesis pathway: the metabolic reactions of MCR and MTA at very high and very low methanol concentrations, respectively.

### Kinetic parameters.

Having identified the rate-determining enzymes and metabolites, we are curious about how they shape the hyperbolic relationship between growth rate and methanol concentration. We first look at specific affinity, *α*, a parameter that defines the initial slope of the hyperbolic relationship. In our case, this parameter is determined by MTA, the enzyme that exerts significant control at very low methanol concentrations, according to the following formula:

(3) $$\alpha =W_{\text{prot}}\cdot \phi_{\text{MTA}}\cdot Y_{\text{P/CH3OH}}\cdot \frac{k_{\text{MTA,app}}}{K_{\text{m,CH3OH}}}$$

where *W*_prot_ is the total protein weight per cell, *Y*_P/CH3OH_ is the protein yield per methanol molecule, *ϕ*_MTA_ is the proteome fraction of MTA, *K*_m,CH3OH_ is the Michaelis constant, and *k*_MTA,app_ is the apparent rate constant, which is the catalytic constant *k*_MTA_ adjusted by the concentration of coenzyme M. The constant *k*_MTA,app_ is determined by the following formula:

(4) $$k_{\text{MTA,app}}=k_{\text{MTA}}\cdot \frac{C_{\text{CoM,0}}}{C_{\text{CoM,0}}\;+\;K_{\text{m,CoM}}}$$

**FIG 6:**
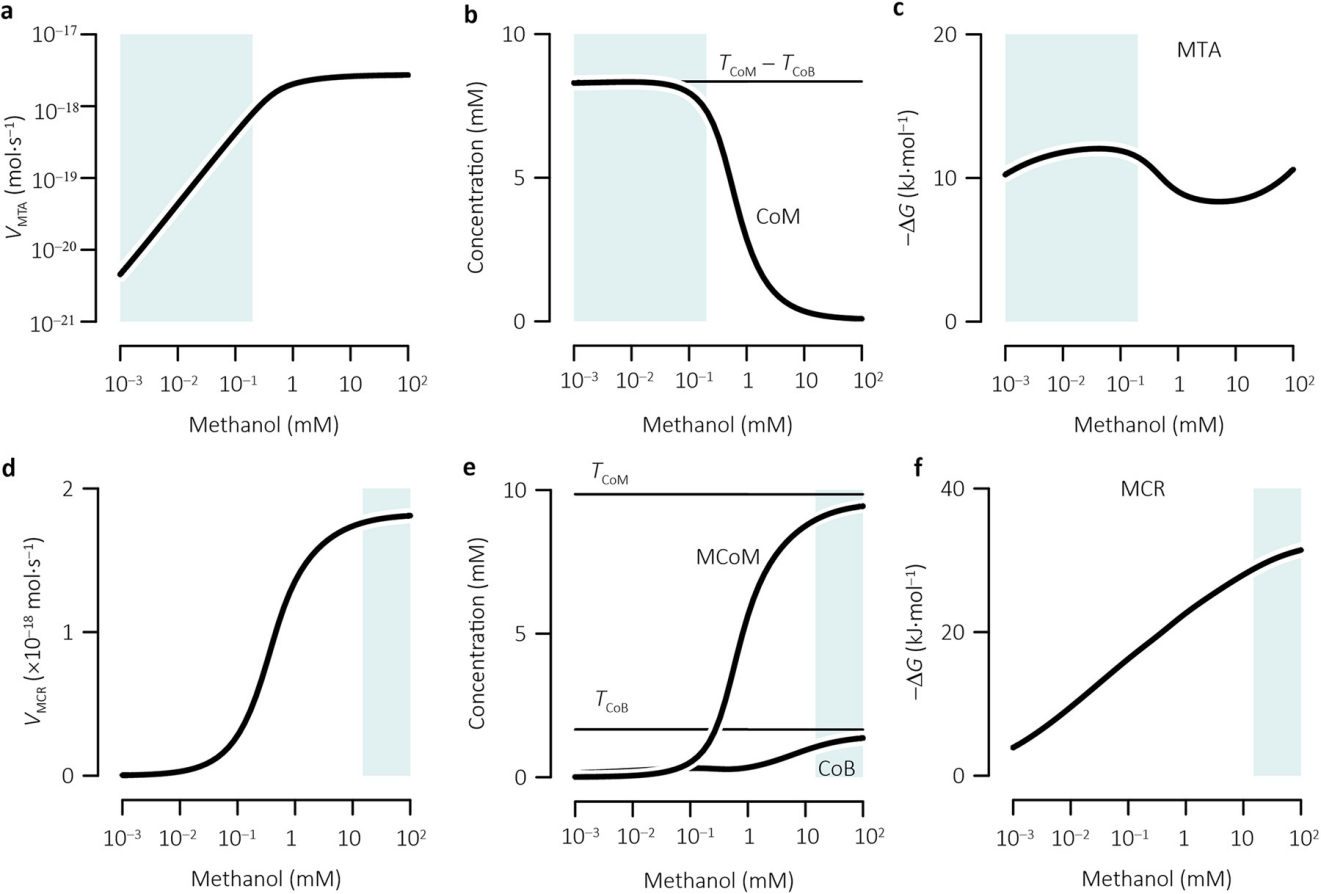
Variations with methanol concentration in the velocity *V*_MTA_ (a), coenzyme M concentration (CoM) (b), and the Gibbs free energy change (Δ*G*) (c) of the MTA reaction, and in the velocity (*V*_MCR_) (d), concentrations of methyl-coenzyme M (MCoM) and coenzyme B (CoB) (e), and the Gibbs free energy change (f) of the MCR reaction. *T*_coM_ and *T*_cob_, concentrations of coenzyme M and coenzyme B moiety, respectively; shaded areas indicate methanol concentrations where growth rate varied linearly (a to c) or approached its maximum (d to f, see Fig. 3a). See the Fig. 1 legend for definitions of abbreviations.

Here, *K*_m,CoM_ is the Michaelis constant and *C*_CoM,0_ is coenzyme M concentration at methanol concentrations near 0. Equation 3 is derived from MCA and the simulation results at <0.2 mM methanol, including that (i) MTA dominates the growth control (Fig. 4a), (ii) growth rate follows first-order kinetics with methanol concentration (Fig. 3a), and (iii) the reaction velocity of MTA varies almost linearly with methanol concentration (Fig. 6a) because coenzyme M concentration stays relatively constant at the concentration difference between coenzyme M and coenzyme B moieties (Fig. 6b), the Gibbs free energy of the MTA reaction falls below −10 kJ · mol^−1^ and does not limit much the reaction velocity (Fig. 6c), and the Michaelis constant (*K*_m,CH3OH_) of methanol is relatively large, i.e., ∼50 mM methanol (62, 63). In equation 3, the product of *k*_MTA,app_ and *ϕ*_MTA_ gives the maximum velocity *V*_MTA,max_ of the MTA reaction, i.e., *V*_MTA,max_ = *k*_MTA,app_⋅*ϕ*_MTA_. Therefore, the specific affinity *α* is a composite parameter that reflects the limitation of methanogen growth placed by MTA and coenzyme M moiety at very low methanol concentrations, and its value varies linearly with the proteome fraction of MTA and hyperbolically with the concentration of coenzyme M moiety.

Maximum growth rate, *μ*_max_, defines the upper bound of the hyperbolic relationship and is related to MCR, the enzyme that significantly controls growth rates at very high methanol concentrations, as shown by the following formula:

(5) $$\mu_{\max}=W_{\text{prot}}\cdot \phi_{\text{MCR}}\cdot Y_{\text{P/CH4}}\cdot k_{\text{MCR,app}}$$

where *Y*_P/CH4_ is the protein yield per methane molecule (g · mol^−1^), *ϕ*_MCR_ is the proteome fraction of MCR, and *k*_MCR,app_ is the apparent catalytic constant (mol · g^−1^ · s^−1^), the catalytic constant *k*_MCR_ adjusted with the concentrations of methyl-coenzyme M and coenzyme B at >15 mM methanol. This is determined by the following formula:

(6) $$k_{\text{MCR,app}}\approx k_{\text{Mcr}}\cdot \frac{T_{\text{CoM}}}{T_{\text{CoM}}\;+\;K_{\text{m,MCoM}}}\cdot \frac{T_{\text{CoB}}}{T_{\text{CoB}}\;+\;K_{\text{m,CoB}}}$$

Here, *T*_CoB_ is the total concentration of coenzyme B moiety. Because coenzyme B moiety has a concentration of 1.7 mM, much larger than the Michaelis constant *K*_m,CoB_ of 59 μM (64, 65), the equation can be further simplified:

(7) $$k_{\text{MCR,app}}\approx k_{\text{Mcr}}\cdot \frac{T_{\text{CoM}}}{T_{\text{CoM}}\;+\;K_{\text{m,MCoM}}}$$

Equation 5 is obtained from the MCA and the simulation results at >15 mM methanol, including (i) growth rate follows a zero-order kinetics with respect to methanol concentration and is controlled primarily by MCR (Fig. 3a and 4a), and (ii) MCR reaction velocity reaches its maximum value (Fig. 6d) because methyl-coenzyme M and coenzyme B approach their maximum possible concentrations (Fig. 6e) and the free energy change of MCR reaction stays below −25 kJ · mol^−1^ (Fig. 6f). Equation 5 suggests that the maximum growth rate, *μ*_max_, is a composite parameter which describes the limitation of methanogen growth by MCR and coenzyme M moiety at very high methanol concentrations, and that its value increases linearly with the proteome fraction of MCR and hyperbolically with the concentration of coenzyme M moiety.

Lastly, the half-saturation constant, *K*_M_, represents the methanol concentration which supports methanogen growth at half of the maximum rate, *μ*_max_. Under this condition, growth rate is controlled by multiple enzymes and chemical moieties (Fig. 4 and 5). Accordingly, the half-saturation constant is determined by the properties of many different enzymes and chemical moieties. Due to the complexity of the metabolic model, no analytical expression is available to relate *K*_M_ to the different enzymes and chemical moieties, and the metabolic significance of *K*_M_ remains unclear.

### Rate laws.

The Monod equation bears a limited link to the metabolic network of methanogen growth. To illustrate this point, we followed Button (66) and Healey (67), approximated the half-saturation constant (*K*_M_) with the ratio of maximum growth rate (*μ*_max_) to specific affinity (*α*), and recast the Monod equation in an alternative form:

(8) $$\mu =\frac{\mu_{\max}\alpha C}{\mu_{\max\;\;}+\alpha C}$$

By combining the equation with the physical meanings of the two parameters (equations 3 and 5), the flux control coefficients of MCR (*ε*_MCR_) and MTA (*ε*_MTA_) can be calculated according to the following formulae:

(9) $$\varepsilon_{\text{MCR}}=\frac{\alpha C}{\alpha C\;+\;\mu_{\max}}$$

and

(10) $$\varepsilon_{\text{MTA}}=\frac{\mu_{\max}}{\alpha C+\mu_{\max}}$$

As shown in Fig. 4a, these two equations capture the overall trends of the control coefficients of MCR and MTA given by the metabolic model, but appreciable deviations appear at >10 μM methanol.

We calculated the flux response coefficient of coenzyme M moiety by substituting equations 3, 4, 5, and 6 to 8. Figure 5a shows that the calculated response coefficient follows the similar trend of the coefficient obtained from the metabolic modeling, but notable gap appears at <10 mM methanol. These results (Fig. 4a and 5a) indicate that there is a mechanistic link between the Monod equation and the metabolic network of methanogen, but the link is limited. In particular, the Monod equation provides an approximation of the control by rate-determining metabolites and enzymes at very low and very high methanol concentrations. However, the equation is missing the remaining enzymes and chemical moieties whose controls are most notable at intermediate concentrations.

By approximately accounting for the rate-determining metabolite and enzymes, the alternative Monod equation should provide an approximation of the kinetics of *M. barkeri* growth. Figure 3 compares the growth rates obtained from the Monod equation and its alternative form to those obtained from the metabolic modeling. By applying the simulation-derived *μ*_max_ and *α* values, the relative difference between the alternative Monod equation and the simulation results remains <5% at <30 μM and >5 mM methanol, and reaches a maximum value of 23% at 0.4 mM methanol. By applying the simulation-derived *μ*_max_ and the *K*_M_ values, the Monod equation reproduces well the simulation results at >0.2 mM methanol, with relative differences of <5%. Below 0.2 mM methanol, the differences become larger at smaller concentrations, reaching a maximum value of 58% at 1 μM methanol. Consistent with these results, the Monod equation fits well with observations of laboratory experiments carried out at relatively large substrate concentrations. For example, Daußmann et al. (49) analyzed the growth rates of *M. barkeri* at methanol concentrations above 0.2 mM, and their results fit well with the Monod equation (*R*^2^ = 0.98, Fig. 3c).

The missing link of the Monod equation suggests that the gap between the simulation results and the Monod equation can be merged by accounting for the enzymes and chemical moieties whose controls are most notable at intermediate concentrations. Considering that the variations in the coefficients of these enzymes and chemical moieties can be described with an asymmetric Gaussian function (Fig. 4 and 5), we suggest calculating growth rate according to the formula below:

(11) $$\mu =\frac{\mu_{\max}\cdot \alpha \cdot C}{\mu_{\max}+\alpha \cdot C}\;+\;\mu_{\text{o}}\exp\left[-\pi {\left(\frac{1}{\beta }\ln\frac{\mu_{\max}}{\alpha C}\right)}^{2}\right]$$

Here, *μ*_o_ is the maximum difference between the rates obtained from the alternative Monod equation (equation 8) and from metabolic modeling (or experimental observations), and *β* is a dimensionless parameter related to the integral breadth of the error peak (Fig. 3d). By fitting the error function to the modeling results, we obtain a best-fit *β* value of 2.1 ± 0.1 at methanol concentration smaller than the ratio of *μ*_max_ to *α*, i.e., *C* < *μ*_max_/*α*, and 3.1 ± 0.2 at C ≥ *μ*_max_/*α*. As illustrated in Fig. 3d, the amended Monod equation matches the simulation results relatively well, with a maximum relative error of less than 5%.

From the perspective of MCA, the Monod equation represents a special case of methanogen growth, where the control coefficients of MCR and MTA are defined by equations 9 and 10, respectively. Likewise, other rate laws in common use are also special cases and their applications to methanogens require different assumptions. For example, the first-order equation

(12) μ=αC

is applicable at < 0.2 mM methanol, where *ε*_MTA_ remains close to 1 and MTA places a dominant control on growth rate (Fig. 3a and 4a). At >15 mM, where MCR dominates the control, the rate expression becomes zero order, i.e., *μ* = *μ*_max_. Moreover, Liebig’s law of the minimum, represented by

(13) $$\mu =\{\begin{array}{cc} \mu_{\max}, & C\;\geq \;C_{\text{o}}\\ \alpha C, & C\;<\;C_{\text{o}} \end{array}$$

is applicable when MCR and MTA are mutually exclusive in regulating methanogen growth; that is, the scaled control coefficient of MCR stays at 1 when methanol concentration is above the concentration *C*_o_, and the coefficient of MTA remains at 1 when methanol concentration is less than the concentration *C*_o_.

## DISCUSSION

We built a kinetic metabolic model for *M. barkeri* growth and analyzed it using metabolic control analysis. We identified the metabolic reactions of MCR and MTA as the rate-determining steps at very high and very low methanol concentrations, respectively, and showed that the Monod equation approximately accounts for control by MCR, MTA, and coenzyme M moiety, but neglects the remaining enzymes and chemical moieties whose controls are most notable at intermediate concentrations. These results support the Monod equation as an approximate rate expression (17–19) and shed new light on microbial kinetics, including how to improve the prediction of growth rates.

In our model, growth rates are computed from first principles based on the stoichiometry, kinetics, and thermodynamics of metabolic reactions, and the resulting hyperbolic growth relationship represents an emergent property of the methanogen metabolic network. Previous studies have approached the hyperbolic relationship by assuming a single rate-determining enzyme, and attributed the relationship to the saturation effect of substrate enzyme interactions as described by the Michaelis-Menten equation (68, 69). Our results suggest that the hyperbolic relationship arises from the substrate-dependent shift in rate-determining metabolic reactions. At the physiological level, this shift manifests as the change in the substrate-growth rate relationship from first-order at very low methanol concentrations to zero-order at very high concentrations, or as a hyperbolic relationship across the entire concentration range.

The results support a new approach to improving the prediction of growth rates. Previous studies have addressed the gap between model and observations by amending the Monod equation with additional parameters and functions, or by using alternative rate expressions, from the logistic equation to the Droop equation (7, 22). Our results cast significant doubt on the effectiveness of these efforts, because a single-term mathematical expression may not be able to properly and fully account for the controls exerted by different enzymes and metabolites and their unique responses to variations of substrate concentrations. Instead, we amended the alternative Monod equation with a Gaussian function to compensate for the incomplete consideration of the growth-rate control. We estimated the parameters of the Gaussian function from the results of metabolic modeling. Alternatively, the parameters can be determined based on the hyperbolic growth relationship obtained from laboratory experiments. The error function improved the application of the Monod equation to methanogen growth, and future tests are required to assess whether the Gaussian function is also applicable to other microbes.

Our results also unify the two views regarding the roles of enzymes in growth rate control. The MCA results quantified the control by individual enzymes in the *M. barkeri* metabolic model, which supports the distribution of control among all network enzymes, a key principle of metabolic control theory (24, 25). At very high or very low substrate concentrations, growth rates are controlled primarily by a single enzyme, which supports the assumption of a rate-determining enzyme (8, 9). Therefore, while metabolic control theory emphasizes the general relationship between growth rates and enzymes across different substrate concentrations, the assumption of a rate-determining enzyme represents special cases at extreme concentrations.

In summary, the Monod equation approximately accounts for the rate-determining metabolic reactions of methanogen growth at very low and very high substrate concentrations. However, the rate law is missing the enzymes and chemical moieties whose controls are most notable at intermediate concentrations. These results support the Monod equation as a useful approximation of growth rates and bring about a fundamental challenge of microbial kinetics: a single-term mathematical expression may not be able to accurately predict growth rates across different substrate concentrations. To improve growth rate prediction, we suggest compensating for the incomplete accounting for growth-rate control by amending the alternative Monod equation with an error function. We also suggest that by integrating the stoichiometry, kinetics, and thermodynamics of metabolic reactions, metabolic modeling can be applied as a numerical tool to delineate the relationship between microbial rates and substrate concentrations and other environmental conditions arising from underlying metabolic mechanisms, moving microbial kinetics beyond the Monod equation and other empirical models.

## MATERIALS AND METHODS

### Kinetic model.

The kinetic model defines the metabolic state of *M. barkeri* using metabolite concentrations and represents methanogen growth as an initial value problem of ordinary differential equations (ODEs). Each ODE describes the rate at which a metabolite concentration changes over time (*t*), and is constructed according to the principle of mass balance. Specifically, the ODE of metabolite j is

(14) $$\frac{dC_{\text{j}}}{\mathrm{dt}}=\frac{1}{V}\cdot \left(J_{\text{j}}\;+\;\sum_{\text{i}}c_{\text{j,i}}v_{\text{i}}\right)$$

where *C*_j_ is the concentration (mol · L^−1^) of the metabolite, *J*_j_ is the diffusive flux (mol · s^−1^) of methanol, CO_2_, or CH_4_, *ν*_i_ is the reaction velocity of enzyme i (mol · s^−1^), *c*_j,i_ is the stoichiometric coefficient of metabolite j in the reaction (negative for metabolite consumption), *V* is the volume of the compartment, which is either the cytoplasm volume (*V*_cyto_) or the membrane volume (*V*_mem_, L). In addition, for coenzyme M, ferredoxin, and other chemical moieties, their concentrations are subject to the law of moiety conservation, as given in

(15) $$C_{\text{M}}=\sum_{\text{i}}C_{\text{M,i}}$$

where *C*_M_ and *C*_M,i_ are the concentrations of total moiety M and its form, i. A special metabolite is the charges, or protons and sodium cations, translocated across the membrane, which contribute to the membrane potential Δψ,

(16) $$\frac{d\Delta \psi }{\mathrm{dt}}=\frac{F}{C_{\text{m}}}\cdot \sum_{\text{i}}c_{\text{C,i}}\cdot \nu_{\text{i}}$$

Here, *F* is the Faraday constant (96,485 C · mol^−1^), *C*_m_ is the membrane capacitance (F, or C · V^−1^), and *c*_C,i_ is the stoichiometric coefficient of protons or sodium cations translocated out of the cytoplasm in the metabolic reaction of enzyme i.

According to Fiksen et al. (70), diffusive flux *J*_j_ (mol · s^−1^) into a cell can be calculated by the following formula:

(17) $$J_{\text{j}}=4\pi D_{\text{j}}r\left(C_{\text{j,env}}\;-\;C_{\text{j,cyto}}\right)$$

where *D*_j_ is the diffusion coefficient (m^2^ · s^−1^), *r* is cell radius and its value is 1 μm (26, 71), and *C*_j,env_ and *C*_j,cyto_ are the concentrations in the environment and the cytoplasm, respectively.

We applied a generalized reversible multiplicative Michaelis-Menten equation to calculate reaction velocity *ν*_i_ (mol · s^−1^) (35, 37),

(18) $$\nu_{\text{i}}=W_{\text{prot}}\cdot k_{\text{i}}\cdot \phi_{\text{i}}\cdot \prod_{\text{S}}\frac{C_{\text{S}}/K_{\text{m,S}}}{1+C_{\text{S}}/K_{\text{m,S}}+C_{\text{P}}/K_{\text{m,P}}}\cdot \left[1\;-\;\exp\left(\frac{\Delta G_{\text{i}}}{\chi_{\text{i}}RT}\right)\right]$$

where *k*_i_ is the catalytic constant of enzyme i (mol · g^−1^ · s^−1^), *ϕ*_i_ is the mass fraction of enzyme i in the proteome, *C*_S_ and *C*_P_ are the concentrations of substrate and product, respectively, *K*_m,S_ and *K*_m,P_ are the respective Michaelis constants, Δ*G*_i_ is the Gibbs free energy change of the reaction (J · mol^−1^), *χ*_i_ is the stoichiometric number of electrons transferred or charges translocated per reaction, *R* is the gas constant (8.3145 J · mol^−1^ · K^−1^), and *T* is the temperature in Kelvin. The free energy change is calculated according to the formula

(19) $$\Delta G_{\text{i}}=\mathrm{RT}\ln\left(\frac{Q_{\text{i}}}{K_{\text{i}}}\right)\;+\;c_{\text{C},\text{i}}F\Delta \psi$$

where *Q*_i_ is the quotient and *K_i_* is the equilibrium constant of the reaction.

We fixed the ATP flux of the maintenance metabolism at 109 $\text{mmol}\cdot {\text{g}}_{\text{dw}}^{-1}\cdot {\text{hr}}^{-1}$ (72), and calculated the fluxes through the pseudo-biomass reaction, and hence the specific growth rate, from the difference between the ATP production flux through ATP synthase and the consumption flux of the maintenance. The results gave a net specific growth rate (30, 73). We also fixed the concentrations of ATP, ADP, and inorganic phosphate in the cytoplasm at 10, 1, and 10 mM (74, 75), assigned the sizes of chemical moiety pools according to the results of previous laboratory analyses, and set dissolved CO_2_ in the laboratory growth media at 20 mM and CH_4_ at 0.1 atm. Further details of the model construction are available in Text S1 in the supplemental material.

### Membrane enzyme concentrations.

We take the proteome fractions *ϕ*_M,i_ of membrane enzymes as decision variables and maximize specific growth rate *μ* according to the formula

(20) $$\max\mu \left(\phi_{\text{M,i}}\right)$$

This optimization is subject to the ODEs of the kinetic growth model (equations 14 and 16; see Text S1), and hence is a dynamic optimization problem. The optimization is further constrained by the total proteome fraction (*ϕ*_M_) of the membrane enzymes, as given by

(21) $$\sum_{\text{i}}\phi_{\text{M,i}}=\phi_{\text{M}}$$

According to laboratory observations (76, 77), we set the value of *ϕ*_M_ at 10%.

### Flux balance analysis.

We estimated the stoichiometric coefficients of the pseudo-biomass reaction by assuming that *M. barkeri* optimizes flux distribution through its metabolic network, including the metabolite fluxes from the methanogenesis pathway to biomass synthesis, in order to maximize growth rate. Accordingly, we analyzed the updated iMG746 genome-scale metabolic model of *M. barkeri* using FBA (39, 72). FBA predicted steady-state flux distribution through metabolic networks from the objective of maximizing growth rates, under the stoichiometric constraints of metabolic reactions and within the permissible ranges of individual fluxes. We drove FBA using methanol uptake flux as input and calculated the stoichiometry of the pseudo-biomass reaction from FBA output, in particular, the specific growth rate and fluxes of ATPs, reduced cofactor F420 and ferredoxin, and acetyl coenzyme A out of the methanogenesis subsystem. We also calculated protein yield, *Y*_P/CH3OH_ and *Y*_P/CH4_ (equations 3 and 5), from the growth rate and the exchange fluxes of methanol and methane.

### Model implementation and analysis.

We implemented and evaluated the kinetic model using the software COPASI (build 217) (78). We performed FBA using the COBRA Toolbox (version 3.0) (79). We followed the method of control vector parametrization and solved the dynamic optimization problem by splitting it into an outer optimization problem and an inner initial value problem (80). The outer optimization problem searches for optimal enzyme levels and is solved with the Nelder-Mead method, a simplex-based direct-search algorithm. The maximum iteration number, tolerance, and relative size of initial simplex were set to 10^4^, 10^−10^, and 10, respectively. The inner initial value problem simulated the dynamics of methanogen growth and was integrated forward for 10^6^ s, well beyond the 10^3^ s required to reach steady-state. Absolute and relative error tolerance were 10^−8^ and 10^−6^, respectively. Because our interest was the growth of *M. barkeri* at constant concentrations of methanol, CO_2_, and CH_4_ in the environment, we focused on steady-state solutions.

According to MCA theory (24, 25), the control exerted by network component i on growth rate can be quantified with the scaled coefficient *ε*_i_, calculated by

(22) $$\varepsilon_{\text{i}}=\frac{\phi_{\text{i}}}{\mu }\cdot \frac{\partial \mu }{\partial \phi_{\text{i}}}$$

where the fractional change in growth rate (*μ*) is divided by a fractional change in the cellular level of the component. For enzymes, *ϕ*_i_ is the mass fraction in the proteome and coefficient *ε*_i_ is the scaled flux control coefficient; for metabolites, *ϕ*_i_ is the concentration and coefficient *ε*_i_ is the scaled flux response coefficient. We numerically calculated the coefficients by changing *ϕ*_i_ by 1%. Coefficients for diffusion reactions were computed by changing diffusion coefficients by 1%.

The kinetic model in SBML and COPASI formats and the MATLAB program for running FBA are available from GitHub (https://github.com/geomicrobiology/Methanosarcina). The model components, including the ODEs and initial concentrations of metabolites, the kinetic expressions of metabolic reactions, and the respective thermodynamic and kinetic parameters, are available in the supplementary material (Data Set S1).
