# Supplementary material for: Limited Mechanistic Link Between the Monod Equation and Methanogen Growth: a Perspective from Metabolic Modeling
Source: Microbiol Spectr. 2022 Mar 3;10(2):e02259-21. doi: 10.1128/spectrum.02259-21 (PMC9045329; doi:10.1128/spectrum.02259-21)
Supplement: SUPPLEMENTAL FILE 2 — Supplemental material. Download SPECTRUM02259-21_Supp_2_seq2.pdf, PDF file, 0.1 MB [file spectrum02259-21_supp_2_seq2.pdf]

Supplementary Information

Supplementary Methods

1. Model Overview

The kinetic model focuses on the methanogenesis pathway of the model methanogen *M. barkeri* (fig 1). It describes the state of the methanogenesis network using the concentrations of 35 metabolites, and simulates the concentration dynamics by considering 21 reactions. Table S1 lists the names of the metabolites. Table S2 lists the stoichiometric equations of the metabolic reactions and the related enzymes. These reactions fall into the following categories:

1. Diffusions of methanol, CO_2, and CH_4 between the environment and the cell;
2. Reactions of 16 methanogenesis enzymes;
3. An ATP hydrolysis reaction by maintenance metabolism; and
4. A pseudo-reaction of biomass synthesis that consumes ATPs, reduced cofactors, and acetyl-coenzyme A.

The model input includes pH, temperature, and the concentrations of methanol, CO_2, and CH_4 in the environment, and the outcome describes the metabolic state of *M. barkeri*, including the metabolite concentrations and how they vary with time and approach a steady state.

2. Compartments and Units

The kinetic model considers three compartments, including the extracellular environment, the membrane, and the cytoplasm. The environment contains the reactants and products of methanogenesis, including methanol, CO_2, and CH_4. The membrane houses membrane-associated metabolites and enzymes, such as methanophenazine and ATP synthase, and stores the electrochemical potential during chemiosmotic energy conservation.

To calculate cytoplasmic volume V_{cyto} and the volume V_{mem} and surface area S_m of the membrane, we assume that the cytoplasm is a sphere with a radius of 1 μm,[1,2] and the membrane has a thickness of 5 nm. To calculate the protein density ρ_{prot}, we assume the dry weight per cell volume at 0.242 $g \cdot cm^{-3}$ and a mass ratio of protein to cell dry weight of 0.63.[3,4] The product of the protein density and the total cell volume gives the protein weight per cell (W_{prot}, eq 2 and 3).

We express metabolite concentrations C_j in mole per liter of the compartment ($mol \cdot L^{-1}$). For example, methanophazine has a unit of mole per liter of the membrane volume, while ferredoxin is in mole per liter of the cytoplasm. We express enzyme abundances in the mass fractions (%) of the total cellular proteins, which can be converted to enzyme mass per cell volume by multiplying with the protein density ρ_{prot}. We express reaction velocities and diffusive fluxes in mol per cell, $mol \cdot s^{-1}$.

3. Initial Value Problem

The kinetic model describes the methanogenesis and growth of *M. barkeri* as an initial value problem – a group of ordinary differential equations (ODEs) combined with the initial concentrations of metabolites. Each ODE gives the rate, dC/dt, at which a metabolite concentration changes with time, and is constructed according to the principle of mass balance, by accounting for the metabolite consumption and production by the metabolic reactions. Table S1 lists the ODEs and the initial concentrations for the metabolites. Table S2 lists the expressions for calculating diffusive fluxes and enzyme reaction velocities.

We assign the initial concentrations of metabolites that share the same moiety on the basis of the cellular moiety concentrations determined by laboratory studies (data sources listed in table S1). We focus on methanogen growth in stable environments and assume that the concentrations of methanol, CO_2, and CH_4 remain constant in the environment.

In addition, to compute the electrical charge stored across the membrane, the model tracks the mole number M of protons and sodium cations translocated out of the membrane. The proton motive force is calculated according to

$$\Delta p = \Delta\psi - \frac{RT\ln(10)}{F}\Delta\mathrm{pH} \tag{S1}$$

where $\Delta\psi$ is the electrical potential difference across the membrane, and ΔpH is the pH difference across the membrane. The electrical potential difference is calculated according to

$$\Delta\psi = \frac{\left(M_{\mathrm{H}^+} + M_{\mathrm{Na}^+}\right)\cdot F}{C'\cdot S_{\mathrm{m}}} \tag{S2}$$

where M is the mole number of protons or sodium cations translocated out of the membrane, and C' is the membrane's specific capacitance (10^{-2} F·m^{2-}).[5] Based on the laboratory observations,[6] we set ΔpH to 0. For the reaction of MTR that translocates sodium cations across the membrane, the electrical potential difference in equation 12 is replaced by the sodium motive force,

$$\Delta s = \Delta\psi + \frac{RT}{F}\ln\left(\frac{C_{\mathrm{Na}^+_{\mathrm{out}}}}{C_{\mathrm{Na}^+_{\mathrm{in}}}}\right). \tag{S3}$$

and by setting the ratio in concentration of sodium cation in the environment ($\mathrm{Na}^+_{\mathrm{out}}$) to sodium cation in the cytoplasm ($\mathrm{Na}^+_{\mathrm{in}}$) at 4.[7,8]

Evaluating enzyme velocity requires the catalytic constant k_{i} and the Michaelis constant ($K_{\mathrm{m,A}}$ and $K_{\mathrm{m,P}}$) of the enzymes. We use the BRENDA database as the starting point,[9] and extract the parameter values from the literature. The values and sources are listed in table S3. To the extent possible, we use the parameter values determined for the enzymes harvested from *M. barkeri* laboratory cultures at exponential growth phase, and in assay media of 37 ºC and neutral pH.[10] Some of the parameters have yet to be determined experimentally, and their values are assigned based on the laboratory analyses of closely-related enzymes.

4. Error Analysis

We quantify the error between the Monod equation and the simulation results using the relative error (RE, %),

$$\mathrm{RE} = 100\% \times \frac{|\hat{\mu}_{\mathrm{i}} - \mu_{\mathrm{i}}|}{\mu_{\mathrm{i}}}. \tag{S4}$$

where $\hat{\mu}_{\mathrm{i}}$ is the specific growth rate at methanol concentration i given by the Monod equation, and μ_{i} is the respective value obtained from the metabolic simulation.

Supplementary Dataset

Dataset S1 (separate file in xlsx). The specifications of the *M. barkeri* metabolic model, including metabolites, their ODEs, and initial concentrations, kinetic expressions of metabolic reactions, their thermodynamic and kinetic parameters, other model parameters, and the laboratory observations for model validation.

Supplementary References

1 Maeder, D. L. *et al.* The *Methanosarcina barkeri* genome: Comparative analysis with *Methanosarcina acetivorans* and *Methanosarcina mazei* reveals extensive rearrangement within *Methanosarcinal* genomes. *Journal of Bacteriology* **188**, 7922-7931, doi:10.1128/jb.00810-06 (2006).

2 Whitman, W. B., Bowen, T. L. & Boone, D. R. in *The Prokaryotes: Other Major Lineages of Bacteria and The Archaea* (eds Eugene Rosenberg *et al.*) 123-163 (Springer Berlin Heidelberg, 2014).

3 Bratbak, G. & Dundas, I. Bacterial dry matter content and biomass estimations. *Applied and Environmental Microbiology* **48**, 755-757 (1984).

4 Gonnerman, M. C., Benedict, M. N., Feist, A. M., Metcalf, W. W. & Price, N. D. Genomically and biochemically accurate metabolic reconstruction of *Methanosarcina barkeri* Fusaro, iMG746. *Biotechnology Journal* **8**, 1070-1079, doi:10.1002/biot.201200266 (2013).

5 White, S. H. A study of lipid bilayer membrane stability using precise measurements of specific capacitance. *Biophysical Journal* **10**, 1127-1148, doi:10.1016/S0006-3495(70)86360-3 (1970).

6 Müller, V., Blaut, M. & Gottschalk, G. The transmembrane electrochemical gradient of Na^+ as driving force for methanol oxidation in *Methanosarcina barkeri*. *European Journal of Biochemistry* **172**, 601-606, doi:10.1111/j.1432-1033.1988.tb13931.x (1988).

7 Peinemann, S., Muller, V., Blaut, M. & Gottschalk, G. Bioenergetics of methanogenesis from acetate by *Methanosarcina barkeri*. *Journal of Bacteriology* **170**, 1369-1372 (1988).

8 Müller, V., Blaut, M. & Gottschalk, G. Generation of a transmembrane gradient of Na^+ in *Methanosarcina barkeri*. *European Journal of Biochemistry* **162**, 461-466, doi:10.1111/j.1432-1033.1987.tb10624.x (1987).

9 Jeske, L., Placzek, S., Schomburg, I., Chang, A. & Schomburg, D. BRENDA in 2019: a European ELIXIR core data resource. *Nucleic Acids Research* **47**, D542-D549, doi:10.1093/nar/gky1048 (2018).

10 Adamczyk, M., van Eunen, K., Bakker, B. M. & Westerhoff, H. V. in *Methods in Enzymology* Vol. 500 (eds Daniel Jameson, Malkhey Verma, & Hans V. Westerhoff) 233-257 (Academic Press, 2011).
